# Individual Differences in Alcohol-Induced Aggression: A Nonhuman-Primate Model

**Published:** 2001

**Authors:** J. Dee Higley

**Affiliations:** J. Dee Higley, Ph.D., is a research psychologist at the National Institute on Alcohol Abuse and Alcoholism, Bethesda, Maryland

**Keywords:** AOD (alcohol or other drug) dependence, Cloninger’s typology, aggressive behavior, AODR (AOD related) violence, impulsive behavior, animal model, serotonin, brain function, cerebrospinal fluid, hydroxyindoleacetic acid, child rearing, predictive factor

## Abstract

Some people are more likely than others to become aggressive after consuming alcohol. Researchers studying alcohol use and aggression hope to identify individual differences in behavior and biochemistry that exist among subjects who become aggressive following alcohol consumption. Research with nonhuman primates has shown that individual differences in brain chemistry predict impulsivity, aggression, and alcohol-induced aggression. These differences appear to be associated with early rearing experiences and remain stable throughout the individual’s life.

Research has demonstrated a consistent relationship between alcohol use and violent behavior. Both perpetrators and victims of violent crimes are likely to have consumed alcohol prior to certain aggressive acts, such as rape, assault, domestic violence, and murder (Collins and Messerschmidt 1993; Arseneault et al. 2000; Cunradi et al. 1999; Scott et al. 1999). For example, in one study of domestic violence, prior alcohol consumption was likely in cases of physical violence but not in cases of verbal aggression, and prior alcohol consumption by both partners was more likely in episodes of severe violence (Leonard and Quigley 1999). Moreover, high alcohol consumption by couples was predictive of future acts of violence by the male partner (Quigley and Leonard 2000). Although not all alcoholics are violent, alcoholics are more likely than nonalcoholics to have a history of violent behavior (Swanson 1993), and alcohol abuse is a major risk factor in spousal violence and homicide (Soyka 1999).

There appears to be a growing consensus that alcohol consumption is related to violent behavior and aggression. In an extensive review of violence-related injuries, Cherpitel (1997) reported that compared with other injured emergency room patients admitted at the same time, people with violence-related injuries entering an emergency room were more likely to have a positive blood alcohol concentration (BAC), to report drinking prior to the injury, and to report more frequent heavy drinking and alcohol-related problems. In addition, recent comprehensive meta-analyses analyzing a high number of studies from different laboratories have concluded that alcohol increases aggression under certain conditions (Bushman 1997; Ito et al. 1996), especially in certain individuals (Zhang et al. 1997).

This article examines the differences in brain chemistry among individuals that influence whether alcohol increases aggression. A nonhuman-primate model is described that has been developed specifically to study these differences and the influence of environment and rearing on brain chemistry and alcohol-induced aggression.

## Individual Differences in Alcohol’S Effect on Aggression

Although it is widely believed that aggression and alcohol use are strongly related, most people who consume alcohol do so without acting aggressively. Predicting which individuals are likely to exhibit aggression following alcohol consumption is an important and intriguing research problem. Perhaps the lack of focus on the individual is one of the reasons why demonstrating a direct causal relationship between alcohol consumption and aggression has, at times, yielded mixed results (see reviews in Brain 1986; Lipsey et al. 1997). Some researchers have suggested that demonstrating a clear relationship between alcohol intake and aggression is difficult, because alcohol consumption increases aggressiveness in some individuals, but decreases it in others (Dougherty et al. 1996; Lipsey et al. 1997; van Erp and Miczek 1997; Winslow et al. 1988; Winslow and Miczek 1985; Zhang et al. 1997). Such mixed findings may be related to researchers’ lack of focus on differences among individuals. For example, research indicates a stronger relationship between alcohol consumption and aggression in subjects with certain traits, including antisocial personality (Moeller et al. 1998), alcohol dependency (Arseneault et al. 2000), impaired cognitive functions (Welte and Wieczorek 1998), previous aggressive episodes (Leonard and Quigley 1999), and low levels of the brain chemical serotonin in the central nervous system (CNS) (Dougherty et al. 1999; Hoaken and Pihl 2000; Pihl et al. 1995; Virkkunen et al. 1995).

## Animal Models and Individual Differences

Studying alcohol-induced aggression in humans has several limitations (see sidebar). One solution to these problems has been to use animal models. Studies with animal models have helped delineate how alcohol affects aggression and the mechanisms that induce these changes. For example, using a rodent model, Miczek and colleagues (1993) showed that alcohol’s effect on aggression is dose-dependent, increasing aggression at low dosages, but decreasing it at higher dosages. This study showed that these effects are not universal, but that wide differences exist between individuals in the effects of alcohol on aggression. The authors found that the same dose of alcohol increased aggression in some subjects, decreased aggression in other subjects, and had no effect on some other subjects. Alcohol’s effect on aggression was found to be a stable, traitlike response (i.e., the effect was consistent for a given individual, like gregariousness is a personality trait that is stable between situations and across time). Whether this difference is present in higher animals, such as primates, is the focus of this article.

**Difficulties in Studying Alcohol-Induced Aggression in Humans**

Studies investigating the effects of alcohol on aggression in humans have typically used an experimental approach in which a person consumes alcohol and is then provoked or asked to compete with another person. Aggression is measured when the person is given an opportunity to shock or verbally threaten a competitor. Under such conditions, many studies have found that aggression is more likely to occur after alcohol consumption (e.g., see Taylor 1993). These studies have outlined a number of possible reasons why alcohol consumption increases the probability of aggression, and a few studies have focused on individual differences and the variables that induce some people to become aggressive (Gustafson 1991*a*, 1991*b*).

Predicting which individuals will become violent after consuming alcohol in real-world settings remains elusive, however. This may be attributable partly to the tenuous relationship between alcohol-induced aggression in the laboratory and real-world alcohol-induced violence. In addition, researchers ethically cannot induce true violence in the laboratory. Such research in humans also poses problems, because difficulties arise in measuring underlying etiological mechanisms, such as variations in the activity of key chemicals in the brain and inherent biochemical differences that exist among people.

—J. Dee Higley

ReferencesGustafsonRAggressive and nonaggressive behavior as a function of alcohol intoxication and frustration in womenAlcoholism: Clinical and Experimental Research158868921991a10.1111/j.1530-0277.1991.tb00620.x1755524GustafsonRMale physical aggression as a function of alcohol, frustration, and subjective moodInternational Journal of the Addictions262552661991b188992410.3109/10826089109058884TaylorSPExperimental investigation of alcohol-induced aggression in humansAlcohol Health & Research World171081121993

### A Nonhuman-Primate Model

Our laboratory has recently used a nonhuman-primate model to investigate individual differences in the effects of alcohol on aggression. Because non-human primates are genetically our closest relatives, findings from these animals are more likely to have relevance to the human condition than findings from other animals. We chose rhesus macaques for our studies because of their close genetic similarity to humans and because they have the most well-characterized CNS of the nonhuman primates. As a consequence of their genetic similarity to humans, rhesus macaques also display similar neurodevelopment and functional neuroanatomy and have many neurobiological features that are similar, if not identical, to human neurobiology (Azmitia and Gannon 1986; Berger et al. 1991; Uylings and van Eden 1990). Rhesus macaques have a similar CNS to humans and, perhaps as a result of this sophisticated CNS, have developed a complex social system. Like that of humans, this social system is based on specific rules for relationships and social behaviors. Moreover, rhesus macaques develop long-lasting social bonds that may endure their life span. For individuals to live in such societies, they must learn to control their impulses—that is, display proper social behavior at appropriate times, in applicable settings, and modify their behavior depending on the situation and partner with whom they are interacting.

In modeling psychopathology, non-human primates offer numerous advantages. Their rearing histories can be controlled systematically and manipulated in a manner not possible in humans, thus allowing researchers to test hypotheses concerning the role of early experiences. Experimental procedures that are not practical in humans are possible in nonhuman primates. For example, researchers often assess the activity of brain chemicals (i.e., neurotransmitters) by taking samples of the fluid that bathes the brain and spinal cord (i.e., cerebrospinal fluid [CSF]). Repeated CSF samples can be readily obtained in a highly controlled fashion to assess subjects longitudinally. An added advantage of using nonhuman primates, such as rhesus macaques, is that the developmental process is compressed; they mature four to five times more rapidly than do humans. Thus, developmental outcomes can be studied prospectively in a fraction of the time it takes to complete a comparable human prospective developmental study. Nonhuman primates are ideal for studying many aspects of human alcohol psychopathology. Nevertheless, some features of human alcohol-related psychopathology exist that non-human primates do not model as well as they do some other features. For example, measuring the role of alcohol expectancies on aggression in the non-verbal primate would be difficult.

## Serotonin and Impulsivity, Drinking, and Violence

Some clues are emerging that may help identify individuals prone to violence when drinking alcohol and reveal the underlying mechanisms involved in this relationship. Not all alcoholics are aggressive. Cloninger (1987, 1988) has proposed that two subtypes of alcoholism exist, type I and type II. Type I alcoholics are believed to consume alcohol primarily to reduce anxiety, whereas alcohol use for type II alcoholics appears to be part of an overall behavior pattern of impulsive, antisocial behavior. Type II alcoholism is, therefore, characterized by impaired impulse control, antisocial traits, difficulties in social relationships, and physically aggressive behaviors (Cloninger 1986, 1987, 1988). Indeed, Bergman and Brismar (1994) concluded that type II alcoholism is determined by a genetic predisposition to both alcoholism and violence.

A principal neurobiological feature of type II alcoholism is a CNS serotonin deficit (Fils-Aime et al. 1996; Javors et al. 2000; Swann et al. 1999; Virkkunen et al. 1994*a*, 1994*b*). Both animal and human studies suggest that reduced serotonin functioning is related to impaired impulse control (Higley et al. 1996*b*, 1996*c*; Linnoila et al. 1983; Mehlman et al. 1994; Soubrié 1986). Therefore, Cloninger and other researchers have suggested that serotonin function is related to loss of control over drinking among type II alcoholics (Cloninger 1986, 1987; Linnoila et al. 1994).

Research also indicates that alcoholics who have reduced levels of serotonin in the brain are prone to violent behavior (Virkkunen et al. 1995, 1994*b*), which may be a product of impaired impulse control. Scott and colleagues (1999), for example, found that perpetrators and victims of assault are more likely to score high in impulsivity and that alcohol further heightens the likelihood of violence among such people. Type II alcoholics, sons of alcoholic fathers (Giancola et al. 1993; Limson et al. 1991; Schulsinger et al. 1986; Sher et al. 1991) and, in some studies, daughters of alcoholic fathers (Sher et al. 1991) have scored high on measures of impulsivity and aggression (Tomori 1994). In a series of studies, Virkkunen and colleagues (1995, 1994*a*, 1994*b*) found that alcoholic men were particularly prone to violence if they had low concentrations of the serotonin metabolite 5-hydroxyindoleacetic acid (5–HIAA). After reviewing such findings, Linnoila and colleagues (1994) concluded that excessive alcohol intake and violence in type II alcoholics may both originate from dysfunctional impulse control, which in turn results from impaired serotonin functioning.

## A Nonhuman-Primate Model for Type II Alcoholism

Nonhuman primates provide a potentially relevant animal model to test the assumptions of Cloninger’s model, which postulates that high levels of violent behavior in alcoholics are associated with impaired serotonin functioning. Like humans, each individual monkey exhibits quantifiable personality traits, and like humans, appropriate early rearing patterns are crucial for normative development. When an alcohol solution is palatable, most nonhuman primates, like humans, will freely consume alcohol. Most subjects consume alcohol at modest levels, but about 20 percent of the subjects will consume alcohol at high levels that produce both visible signs of intoxication and blood alcohol levels above 0.10 percent. During the past decade, we have investigated individual differences in alcohol consumption in nonhuman primates by studying CNS and temperamental influences on excessive alcohol consumption. In this effort, we have focused on the relationships between neurophysiology, impulsivity, aggression, and alcohol consumption.

Paralleling studies in humans, research with nonhuman primates has shown that low CSF 5–HIAA concentrations are associated with type II-like behaviors, including behaviors characteristic of impaired impulse control (Higley and Bennett 1999). For example, nonhuman primates with low CSF 5–HIAA concentrations exhibit spontaneous, long leaps at dangerous heights and are repeatedly captured in baited traps (Higley et al. 1996*b*, 1996*c*; Mehlman et al. 1994). Low CSF 5–HIAA concentrations in nonhuman primates also are correlated with increased rates of wounding, unprovoked and unrestrained violence, and inappropriate aggression (Higley et al. 1996*a*, 1996*b*, 1996*c*, 1992*a*; Mehlman et al. 1995, 1994; Westergard et al. 1999).

Research findings suggest that low CSF 5–HIAA concentrations are not correlated with high rates of overall levels of aggression, but only are correlated with high levels of spontaneous, impulsive aggression, which tends to escalate to physically damaging conflicts (Higley et al. 1996*c*). This association indicates that the high rates of violent aggression shown by these monkeys probably result from impaired impulse control. Furthermore, escalated aggression in subjects with low CSF 5–HIAA concentrations is strongly correlated with measures of impulsivity (Higley et al. 1996*b*). In addition, alcohol consumption is binge-like and excessive in subjects with low CSF 5–HIAA concentrations (Higley et al. 1996*e*).

Research using nonhuman primates has been particularly useful, because it has delineated possible underlying mechanisms that produce impaired serotonin functioning. Long-term studies of non-human primates in which researchers have repeatedly sampled CSF from the same subjects have shown that individual differences in CSF 5–HIAA concentrations remain stable over time (Higley et al. 1992*b*, 1993; Kraemer et al. 1989). Furthermore, even under conditions when the settings and situations change, individual differences remain stable. For example, when CSF 5–HIAA is sampled while the subjects live alone in single cages, and then again after they are placed into new social groups, individual differences in CSF 5–HIAA concentrations are positively correlated (Higley et al. 1996*a*). Furthermore, when 14 CSF samples were obtained over a 1-year period from the same adult female subjects, individual differences in the 5–HIAA levels remained consistent over the time period (i.e., the levels had high interindividual stability) (Higley et al. 1996*a*).

This consistent high level of interindividual stability is not limited to the laboratory setting, where environmental changes are closely controlled and experiences are relatively homogeneous. In their natural environment, adolescent male macaques migrate from their birth groups to join new social groups (Higley et al. 1994). This is a period of high social stress, because the young males must form new relationships and face social challenges in which trauma and premature mortality are relatively frequent (Higley et al. 1994). Despite these rather dramatic environmental changes, interindividual differences in CSF 5–HIAA concentrations during the year preceding migration to a new social group are positively correlated with interindividual differences in CSF 5–HIAA concentrations following migration. Such interindividual differences also appear to stabilize beginning early in life. CSF 5–HIAA concentrations were stable when samples were taken from monkeys 14, 30, 60, 90, 120, and 150 days after birth (Shannon et al. 1995). These early differences remain stable, with mean concentrations of CSF 5–HIAA taken in late infancy (i.e., at age 6 months) predicting concentrations 1 year later in middle childhood (Higley et al. 1992*b*) and into adulthood (Higley et al. 1996*d*, 1996*e*). Such findings suggest that as a potential risk factor, low CSF 5–HIAA concentrations are present early in life and endure over time and across situations.

## Influence of the Early Environment on CNS Development

The relationships between serotonin and behavior problems may begin early in life. In one study, pubertal children of alcoholics who exhibited low concentrations of serotonin in their blood received high ratings for behavioral disinhibition and aggression (Twitchell et al. 1998, 2000). Similarly, children with high ratings on measures of aggression and social deviancy and low ratings on competent social behaviors displayed relatively low CSF 5–HIAA concentrations (Kruesi et al. 1990). Early events may adversely affect the serotonin systems.

Animal studies have shown that appropriate environmental input during developmental periods is essential for the normal development of the CNS (Black et al. 1989; Greenough 1987). Primate societies are explicitly structured to assure that infants receive such input. When the primate order diverged into Old and New World species, New World species evolved a new system of parental care not typically seen in Old World species. Among most Old World monkey societies, newborns initially develop their social skills within the protective and watchful tutelage of their biological mothers. Mothers are especially important social agents through which infant and juvenile monkeys develop the capacity to properly inhibit and express emotions, including aggression (Bernstein and Ehardt 1986; Harlow 1969; Harlow and Harlow 1965; Higley and Suomi 1986, 1989).

In research settings, instances in which peers are the primary teachers of young monkeys (peer-only rearing) have been widely used to study development in monkeys. The subjects are removed from their mothers at birth and reared without adults but with constant access to other age-matched infants. Peer-only reared monkeys exhibit many deficits in impulse control. For example, as adolescents, when alcohol is freely available, they are prone to excessive alcohol consumption (Higley et al. 1991*a*), and during competitive interactions, minor episodes of aggression are more likely to escalate to severe aggression (Higley et al. 1994).

Interaction between young monkeys and adults not only affects the acquisition and development of behavior, but also plays a crucial role in the organization and proper development of the CNS. For example, a number of studies using nonhuman primates have shown that early experiences affect serotonin functioning during infancy and childhood (Higley et al. 1992*b*, 1993, 1996*e*; Kraemer et al. 1989). These studies have shown that adult influence, particularly maternal input, is critical to the development of the CNS serotonin system. In the absence of adult influence, the development of serotonin functioning is impaired. When CSF 5–HIAA was obtained from peer-only reared and mother-reared monkeys 14, 30, 60, 90, 120, and 150 days after birth, parentally neglected, peer-reared subjects exhibited lower CSF 5–HIAA concentrations than did mother-reared subjects (Shannon et al. 1995). One study with a limited sample size suggested that the effect of early rearing experiences on CSF 5–HIAA may disappear by adolescence (Higley et al. 1991*b*). However, in a study in which peer-only reared and mother-reared subjects were studied from infancy into adulthood, the peer-only reared subjects exhibited lower CSF 5–HIAA concentrations than did the mother-reared subjects in both infancy and adulthood (Higley et al. 1996*e*).

Although conclusions may be somewhat preliminary, recent studies indicate that early adverse experiences may affect human as well as nonhuman-primate CNS serotonin functioning. Boys reared in adverse environments, such as with parents who are absent or who show harsh parenting practices, show lower levels of a molecule that binds with serotonin and mediates its activity (i.e., a serotonic receptor) compared with boys reared in better family settings (Pine et al. 1996). In a recent study of adults who had grown up in and were currently living in poverty, researchers used a serotonin-acting drug to stimulate the release of the hormone prolactin and found that these adults released less prolactin than did people reared in better environments (Matthews et al. 2000). Therefore, some evidence indicates that as with nonhuman primates, being brought up in a less favorable environment may adversely affect CNS serotonin functioning in humans.

## Alcohol and Aggression in Nonhuman Primates

Some studies of nonhuman primates have shown that alcohol increases the probability of aggression in some individuals (Winslow and Miczek 1988). Evidence shows that alcohol is more likely to increase aggression in subjects with impaired CNS serotonin functioning. For example, we found that CSF 5–HIAA concentrations were negatively correlated with high ratings for lifetime aggressiveness (Doudet et al. 1995)—that is, animals with low CSF 5–HIAA concentrations were more likely to act aggressively throughout life. Moreover, whereas nonhuman primates with low CSF 5–HIAA concentrations are typically more likely than others to consume alcohol in excess and exhibit severe aggression, the administration of sertraline, a compound that interferes with serotonin’s activity (i.e., a serotonin reuptake inhibitor), reverses these aberrant behaviors by reducing both alcohol consumption and aggression (Higley et al. 1998). Similarly, administration of the serotonin precursor tryptophan blocks self-aggression in primates prone to self-abuse (Weld et al. 1999).

Nevertheless, although low CSF 5–HIAA concentrations are correlated with both excessive alcohol consumption and impulsive, violent behavior (e.g., see Higley et al. 1996*c*, 1996*d*, 1996*e*; Mehlman et al. 1994), no long-term studies have been conducted on the relationships between serotonin, a life history of violent behavior, and alcohol-induced aggression. Based on the above studies, we posited that low CSF 5–HIAA concentrations would correlate with high lifetime rates of violent behavior and that a lifetime pattern of violent behavior prior to alcohol exposure would predict aggressive behavior under the influence of alcohol. Such a finding would indicate that individuals who act aggressively under the influence of alcohol have a life-long pattern of aggression, and that in these individuals, alcohol probably increases the likelihood of acting aggressively.

To test this theory, monkeys were given alcohol intravenously to produce modest levels of intoxication (i.e., a blood alcohol concentration of about 0.25 percent). Then each animal was placed into a separate room, and researchers scored each monkey’s incidents of aggressive behavior toward a provoker. To obtain these scores, an investigator entered the room, stood in the opposite corner from the monkey, maintained eye contact, and directed an open-mouth threat face at the monkey every 30 seconds for 5 minutes, a procedure shown to elicit mild aggression from macaques (Kalin and Shelton 1989). To test the relationship between lifetime rates of aggression and aggression during intoxication, two researchers who had extensive experience observing rhesus monkey behavior independently rated the animals for aggressiveness using the monkeys’ medical treatment records. These ratings were based on the frequency of removal from the home cage for perpetrating violent behavior or receiving veterinary care for aggression and the frequency and severity of wounds resulting from aggressive encounters.

Animals rated as having a greater lifetime history of severe physical aggression were more likely than others to exhibit higher rates of aggression while intoxicated. Day-to-day home-cage competitive aggression—which is used to defend status, tends to be controlled and restrained, and generally does not result in injury—was not correlated with aggression during intoxication. A life history of severe traumatic, but not restrained, competitive aggression was also predictive of aggression when the animals were intoxicated. Studies investigating humans also show that alcohol is most likely to increase aggression in people already disposed to violent behavior (Zhang et al. 1997).

Both animal and human studies show that rates of aggression during intoxication are stable within individuals (Miczek et al. 1992; Zhang et al. 1997). Similarly, Jaffe and colleagues (1988) found that men who exhibit aggressive behavior when intoxicated are more likely to have had high levels of aggression as children. Our findings suggest that high rates of aggression during intoxication are an extension of a life-long pattern of impulsive, violent aggression, rather than a special form of aggression. This finding is similar to what has been found among men and nonhuman primates with low CSF 5–HIAA concentrations. These studies show that impulsive, severe aggression, but not controlled or competitive aggression, is correlated with diminished serotonin functioning (Coccaro 1989; Higley et al. 1996*b*; Linnoila et al. 1983). It is noteworthy that in a study of men selected at random from the general population, reducing circulating levels of the serotonin precursor tryptophan augmented rates of alcohol-induced aggression, particularly under conditions of provocation (Pihl et al. 1995).

## Conclusions and Future Directions

As in humans, primates with low CNS serotonergic activity exhibit behaviors indicative of impaired impulse control and unrestrained aggression. The studies reviewed here suggest that impaired serotonin functioning may increase the risk for aggression following alcohol consumption. Possibly, a common neurobiological mechanism, such as low serotonin production and turnover, underlies both excessive alcohol consumption and impulsive aggressive behavior. The reviewed studies also show that interindividual differences in the CNS serotonin turnover rate exhibit traitlike qualities and are stable across time and settings, with day-to-day individual differences in CSF 5–HIAA concentrations, for example, showing positive correlations. Such findings suggest that to the extent that type II psychopathology is mediated by an impaired CNS serotonin system, subjects with low CSF 5-HIAA concentrations may have a long-term risk for type II psychopathology and alcohol-mediated violence that can be detected early in life. Maternal and paternal genetic influences play major roles in producing low CNS serotonin functioning beginning early in life. These genetic influences on serotonin functioning are exacerbated by early rearing experiences, particularly parental deprivation, thus affecting the risk for type II alcohol psychopathology.

Whereas impulsivity is thought to underlie the aggressive tendencies in serotonin-deficient, type II alcoholics, little is known about other cognitions that are associated with serotonin deficits. It would not be surprising to find that alcohol affects other cognitive skills that are involved in controlling aggression. Studies are under way to assess the extent to which cognitive deficits in nonhuman primates with low CSF 5–HIAA concentrations are limited to measures of impulsivity or result in a larger overall pattern of cognitive deficits. Moreover, temperaments that accompany subjects with low CSF 5–HIAA concentrations may play a role in the emotional instability that often is seen in humans with low CSF 5–HIAA concentrations. Finally, serotonin has received much attention in this area of research. Clearly, other neurotransmitters are involved in regulating aggression and alcohol consumption. Subsequent studies are under way to assess their roles together with serotonin in controlling ntrollin aggression mediated by alcohol.
